# Anticancer activity of glycoalkaloids from *Solanum* plants: A review

**DOI:** 10.3389/fphar.2022.979451

**Published:** 2022-12-07

**Authors:** Magdalena Joanna Winkiel, Szymon Chowański, Małgorzata Słocińska

**Affiliations:** Department of Animal Physiology and Developmental Biology, Institute of Experimental Biology, Faculty of Biology, Adam Mickiewicz University, Poznań, Poland

**Keywords:** *Solanaceae*, glycoalkaloids, *Solanum* plants, natural compounds, antitumor potential, cancer, molecular mechanisms, apoptosis

## Abstract

Cancer is still one of the main causes of death worldwide. For this reason, new compounds that have chemotherapeutic potential have been identified. One such group of substances is *Solanaceae* glycoalkaloids (GAs). They are natural compounds produced by plants widely used in traditional medicine for healing many disorders. Among others, GAs exhibit significant antitumor properties, for example, a strong inhibitory effect on cancer cell growth. This activity can result in the induction of tumor cell apoptosis, which can occur *via* different molecular pathways. The molecular mechanisms of the action of GAs are the subject of intensive research, as improved understanding could lead to the development of new cancer therapies. The genetic basis for the formation of neoplasms are mutations in protooncogenes, suppressors, and apoptosis-controlling and repair genes; therefore, substances with antineoplastic properties may affect the levels of their expression or the levels of their expression products. Therapeutic compounds can be applied separately or in combination with other drugs to increase the efficiency of cancer therapy; they can act on the cell through various mechanisms at different stages of carcinogenesis, inducing the process of apoptosis, blocking cell proliferation and migration, and inhibiting angiogenesis. This review summarizes the newest studies on the anticancer properties of solanine (SN), chaconine (CH), solasonine (SS), solamargine (SM), tomatine (TT) and their extracts from *Solanum* plants.

## Introduction

Cancer is one of the most common causes of death globally. In 2020, approximately 19 million new cases of cancer and 10 million deaths due to cancer were registered. The number of cancer cases is expected to increase to more than 28 million over the next 20 years (Sung et al., 2021). In men, the most common neoplasms were lung cancer (14.3%) and prostate cancer (14.1%). In women, breast (24.5%) and colorectal cancer (9.4%) are the most frequently diagnosed. In 2020, the highest percentage of cancer deaths in men was caused by lung cancer (21.5%) and liver cancer (10.5%), while in women, breast cancer (15.5%) and lung cancer (13.7%) were the leading causes of death (Sung et al., 2021). These estimations are based on the GLOBOCAN database prepared by the International Agency for Research on Cancer.

Why is cancer so widespread among people worldwide? The cells of our body grow and multiply during life as a result of division processes that run according to the information contained in the genetic material. The cell cycle of healthy cells stops at the right time. New, young cells replace stem cells, which age and undergo apoptosis. All cell divisions are followed by exact replication of genetic material, resulting in two identical DNA molecules. However, it is possible for various errors to occur in this process. These mistakes can appear spontaneously and may be induced by carcinogens or inherited from parents (Cooper, 2000; Sarkar et al., 2013). These errors lead to mutations in the genetic material and, as a consequence, damaged cells may grow and reproduce in an uncontrolled manner. In a healthy organism, there are many mechanisms of tumor suppression. For example, they enable the cell cycle of mutated cells to be stopped. However, if the repair mechanisms do not work properly, a tumor can develop. With age, these systems function less and less efficiently; in addition, DNA damage accumulates in cells during life; therefore, the risk of developing tumors increases over time (Cooper, 2000; Kamal et al., 2022).

The mechanisms of tumor formation are complex, but the direct causes are mutations in genes encoding proteins crucial for the proper course of cell division, such as protooncogenes (stimulating cell proliferation), suppressor genes (inhibiting cell proliferation), genes controlling cell apoptosis, and repair genes. Protooncogenes include genes that encode growth factors and their receptors, messenger proteins, transcription factors, and cell cycle regulators. Mutations may change the structure or quantity of proteins and thus affect the physiological function they perform in a healthy organism (Domagala, 2007; Sarkar et al., 2013; Kamal et al., 2022).

Unlike normal cells, mutant cells divide excessively in an uncontrolled manner and do not fulfill the role of their origin. Benign tumor cells usually respond well to treatment because they are localized in the tissue, e.g., lipoma in fat tissue. However, when tumor cells can travel *via* the blood or lymphatic system to other parts of the body, we call it a cancer that is a threat to the proper functioning of the body.

Currently, the main method of treating malignant tumors is chemotherapy, a method based on the use of cytostatic drugs that inhibit cell division due to DNA damage or cell cycle inhibition. These drugs are not very specific and damage healthy, rapidly dividing cells such as the bone marrow and hair follicles, causing a number of side effects such as anemia and baldness. Many patients undergo radiation therapy, which results in the production of free radicals that destroy cell DNA. Hormone therapy is used to treat tumors whose growth depends on the stimulating or inhibitory action of certain hormones to produce an antitumor effect, e.g., in breast and thyroid cancer (National Cancer Institute, 2017). Neoplastic lesions can be surgically removed with a margin of healthy tissue. Most modern cancer treatments include immunotherapy and molecularly targeted drugs. In 2018, the Nobel Prize for Physiology or Medicine was awarded to James Allison and Tasuku Honjo for developing cancer immunotherapy based on stimulating the immune system to recognize and destroy cancer cells, e.g., by giving a vaccine. The targeted drugs are monoclonal antibodies or kinase inhibitors, whose action is based on inhibition of the molecular mechanisms of tumor formation and growth (National Cancer Institute, 2017).

Searching for new methods in cancer treatment is critical for global cancer control. It is important to remember that plants through millions years of evolution developed and produce an enormous number of biologically active substances. That is why modern medicine often reaches for achievements of traditional knowledge about medicinal plants built over the years. The first antimalarial drug was obtained from *Cinchona* spp. Artemisinin, lactone with antimalarial properties, was isolated from *Artemisia annua* L. The source of morphine was *Papaver somniferum* L. There are much more examples of medicinal plants usage (Pirintsos et al., 2022). *Solanaceae* plants are also rich in bioactive metabolites such as alkaloids, glycosides and lignans. Antimicrobial, insecticidal and antiinfectious properties of *Solanum* plants were used traditionally, for example, pepper (*Capsicum annuum* L.) was applied to prevent cold and improve digestion, and potato (*Solanum tuberosum* L.) in case of burns, cough, and spasms (Afroz et al., 2020). Local inhabitants of rural areas around the world have comprehensive traditional knowledge about the use of *Solanaceae* as medicinal plants for cancer treatment (Khan et al., 2021). However, the path from the traditional ethnopharmacology to discovery of botanical drugs and development a specific method of treatment is not easy to follow, but early descriptions of therapeutic properties of natural plant products can indicate potential application of such compounds in medicine and facilitate developing effective drugs. Moreover, medicinal plants are still often used as an alternative to modern medicine or as primary remedies in developing countries (Vandebroek, 2013; Pirintsos et al., 2022).

Many compounds of plant origin show anticancer properties, such as components of *Solanum nigrum* L. fruit. They were identified, and numerous cancer-related targets of their action were recognized based on the molecular docking method (Yang et al., 2021). For example, alkaloids, which are alkaline substances with high biological activity that contain a nitrogen atom in their structure, are currently used in cancer therapy. *Vinca* L. alkaloids (vinblastine and vincristine) and yew (*Taxus baccata* L.) alkaloids (paclitaxel) act by binding to microtubules that form the mitotic spindle, resulting in inhibition of cell division. Vinblastine and paclitaxel are used in the treatment of breast and lung cancer, and vincristine is used in the treatment of acute lymphoblastic leukemia (Pantziarka et al., 2021). Another compound with anticancer properties is camptothecin, a quinoline alkaloid of *Camptotheca acuminata* Decne. a topoisomerase I inhibitor. It was withdrawn from clinical trials due to its high toxicity and low water solubility; however, derivatives of this alkaloid are used in the treatment of ovarian and lung cancer (Gurgul and Litynska, 2017).

**FIGURE 1:**
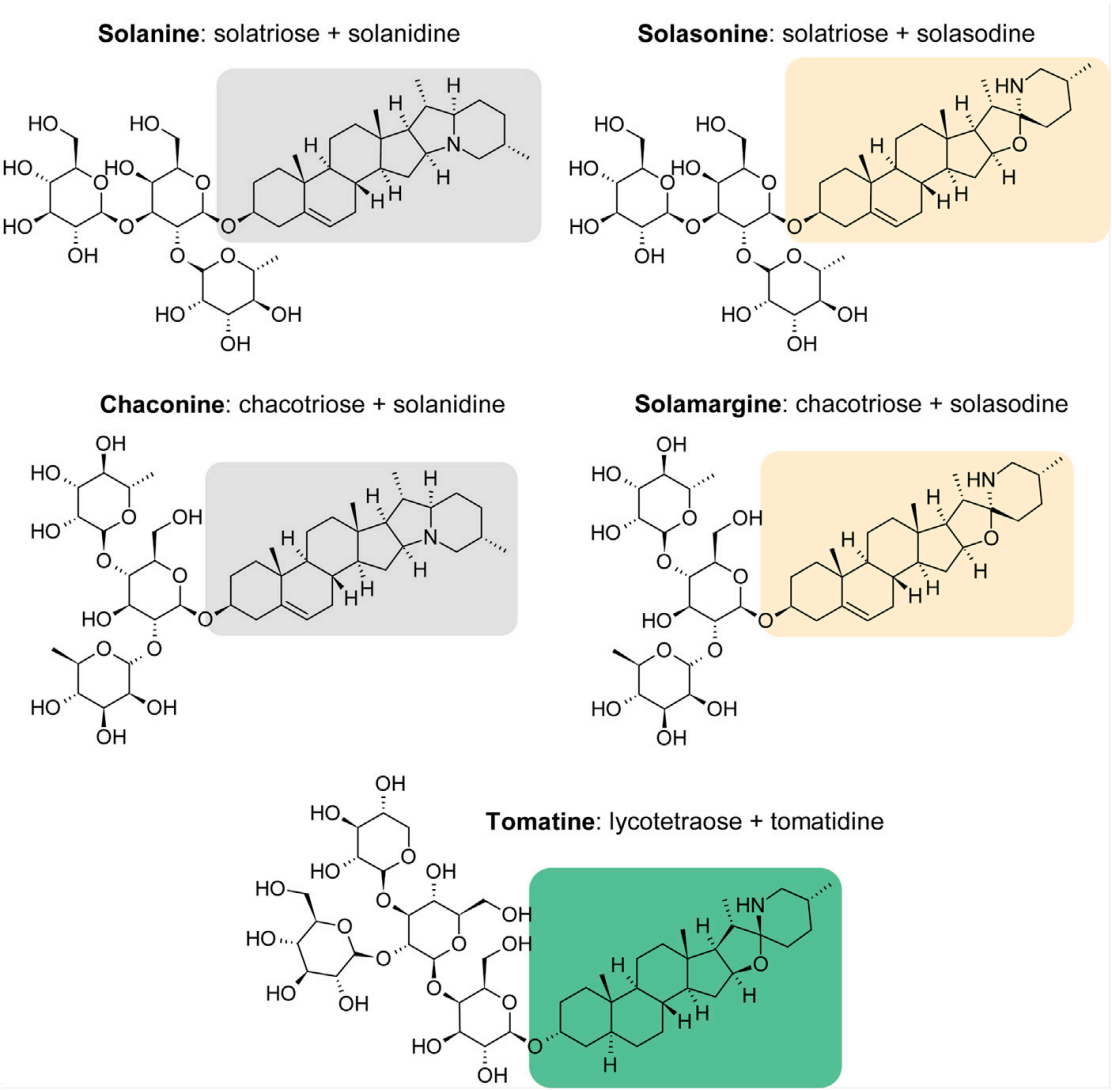
Structures of *Solanaceae* glycoalkaloids.

Glycoalkaloids (GAs), or alkaloids with an attached sugar group, also exhibit toxic properties against cancer cells. One group of plants that is rich in GAs are the nightshades (*Solanaceae*). They are common in the world and comprise many useful plant species, i.e., potato *Solanum tuberosum* L., tomato *Solanum lycopersicum* L., black nightshade *S. nigrum* L. and sweet pepper *Capsicum annuum* L. Nightshade plants are a source of many biologically active substances, including alkaloids. For example, atropine present in deadly nightshade (*Atropa bella-donna* L.) acts as an antagonist of muscarinic receptors, and nicotine occurring in tobacco (*Nicotiana tabacum* L.) is an agonist of N-acetylcholine nicotinic receptors (Gopalakrishnakone et al., 2017). GAs are secondary metabolites that are sugar derivatives of steroidal alkaloids. GAs have an amphiphilic nature and are composed of a hydrophilic carbohydrate chain attached to the 3-OH position and the aglycone part, which is a 27-carbon skeleton of cholastane with a nitrogen atom in the ring structure. Solanine (SN) and chaconine (CH) are composed of an aglycon called solanidine, the aglycon part of solasonine (SS) and solamargine (SM) is solasodine, and the structure of tomatine (TT) is based on the tomatidine moiety (Milner et al., 2011). The chemical structures of the GAs that are the subject of this study are shown in Figure 1. Most GAs are solids, less frequently liquids, and are well soluble in organic solvents, with high biological activity. These compounds are produced in flowers, leaves, roots, and sprouts. For example, the source of SN is the potato tuber, whereas TT is found in the leaves of tomatoes. GA synthesis in plants occurs during seed germination and is most intense during the flowering period. As a result of exposure to light, potato tubers turn green, and the SN content increases tenfold, as the synthesis of the steroid GAs is related to the biosynthesis of chlorophyll (Friedman, 2006).

The molecular mechanisms underlying GA action are the subject of intensive research, as improved understanding could lead to the development of new cancer therapies. The aim of this study is to review the latest research on the anticancer properties of *Solanaceae* GAs. Studies in English from 2015 to 2022 were reviewed on the Web of Science using the keywords *Solanaceae*, glycoalkaloids, solanine, chaconine, solasonine, solamargine, tomatine, tumor, cancer and their combinations.

## Toxicity of *Solanaceae* glycoalkaloids

In plants, alkaloids are a natural defense against phytopathogens and herbivores. Pure substances and their plant extracts show dose-dependent toxicity against fungi, insects, and other organisms (Boulogne et al., 2012; Chowanski et al., 2016; Chowanski et al., 2018; Spochacz et al., 2018), and have been used in the production of pharmaceuticals due to their properties (Oksman-Caldentey, 2007). Because ‘only the dose makes a substance a poison’, alkaloids can be therapeutic as well as toxic.

In *in silico* studies, the toxicity of solanidine, solasodine, SN, SS and SM was calculated and compared with the toxicity of doxorubicin hydrochloride (an anticancer drug) and tetracycline. The analysis predicted that the mentioned GAs do not cause any risk of mutagenic, tumorigenic, or irritant toxicity, although most of them exhibit a mild undesired impact on the reproductive system (Ahmad, 2019). SN influenced oocyte maturation and disrupted embryonic development in a pig model (Lin et al., 2018) and exhibited cytotoxic activity in TM3 and TM4 mouse testis cell lines (Park et al., 2019). SN at a concentration of 20 µM and higher also showed cytotoxicity in human trophoblast cells of the HTR-8/SVneo line. SN increases the expression of autophagic genes, triggers autophagosomal generation, and stops the cell cycle in the S and G2/M phases. By activating apoptosis and autophagy, it inhibits cell viability, migration, invasion, and blood vessel formation (Chen et al., 2021). SN and CH have also been hypothesized to contribute to the formation of congenital abnormalities of the nervous system, consisting of neural tube defect (NTD) formation and disturbances in closure during fetal development; however, there is no evidence of their action in humans (Cuschieri and Calleja-Agius, 2020).

SS and SM show cytotoxic effects in the normal cell lines L-02, BEAS-2B, HK-2, and MCF-10A (Li et al., 2016). Hepatic toxicity and downregulation of *cyp450* genes were found in mice treated with SS, and the toxicity depended on the genetic background (Zhong et al., 2018). The IC_50_ of SS was higher in AGS and HT-29 cells than in healthy cells (Akter et al., 2015). SM from *S. nigrum* L. fruits exhibited cytotoxic effects in MGC803, HepG2, and SW480 cancer cell lines; however, the LD_50_ values were higher than those of the positive control KPT330 (Gu et al., 2018). SM at a concentration greater than 14.2 g/ml and SS greater than 28.8 g/ml showed cytotoxicity in V79 cells. However, these GAs do not cause genotoxic effects or aberrations of the chromosome. They cannot reduce the genotoxic effects of camptothecin and etoposide, but SM and SS decreased DNA damage and the prevalence of chromosomal aberrations caused by methyl methanesulfonate, an alkylating compound (Munari et al., 2022). Furthermore, treatment with SM or SM in nanoparticles of yttrium vanadate functionalized with 3-chloropropyltrimethoxysilane at doses of 5 or 10 mg/kg/day administered subcutaneously for 5 days did not show any apparent systemic toxic effects, nephrotoxicity, or genotoxicity in mice. Furthermore, there was less damage to liver DNA caused by melanoma model B16F10 cells in animals treated with 10 mg/kg/day SM (Furtado et al., 2022). SM exhibited only a slight effect on healthy primary bovine aortic endothelial cells (BAECs), rat fibroblasts, and epithelial cells (Al et al., 2016), and the cytotoxic effect of SM and SN on the NIH3T3 and VERO healthy cell lines was smaller than that on the MCF-7, MDA-MB-231, AGS, and HT-29 cancer cell lines (Akter et al., 2015). The extract of the *S. nigrum* L. fruit exhibited low cytotoxicity (Yang et al., 2022), while there was no toxic effect in mice orally treated with extracts of *Solanum capsicoides* All. seeds (2000 mg/kg) (Petreanu et al., 2016).

The effects of different concentrations of TT on gene expression and cell monolayer integrity were also studied. Research showed that TT at concentrations lower than 20 μg/ml was safe in the human colon cell line Caco-2. This compound does not affect monolayer integrity or metabolism and does not induce apoptosis or impact the cell cycle; however, it influences cytokine-mediated signaling genes (Arena et al., 2018). The toxicity of the methanolic extract of tomato leaves was evaluated in rats and did not show any hepatotoxic effects even at the highest dose (1,000 mg/kg b.w.) after 28 days of treatment but influenced lipid metabolism and exhibited immunostimulatory effects (Nguenang et al., 2020). Furthermore, the extract of TT from green tomatoes showed a cytotoxic effect on cancer and normal cells. In breaker-stage vegetables, the action of this GA is believed to be masked by antioxidant substances (Del Giudice et al., 2015). For these reasons, special caution should be considered during the study of the pharmaceutical use of GAs (Li et al., 2016).

**FIGURE 2:**
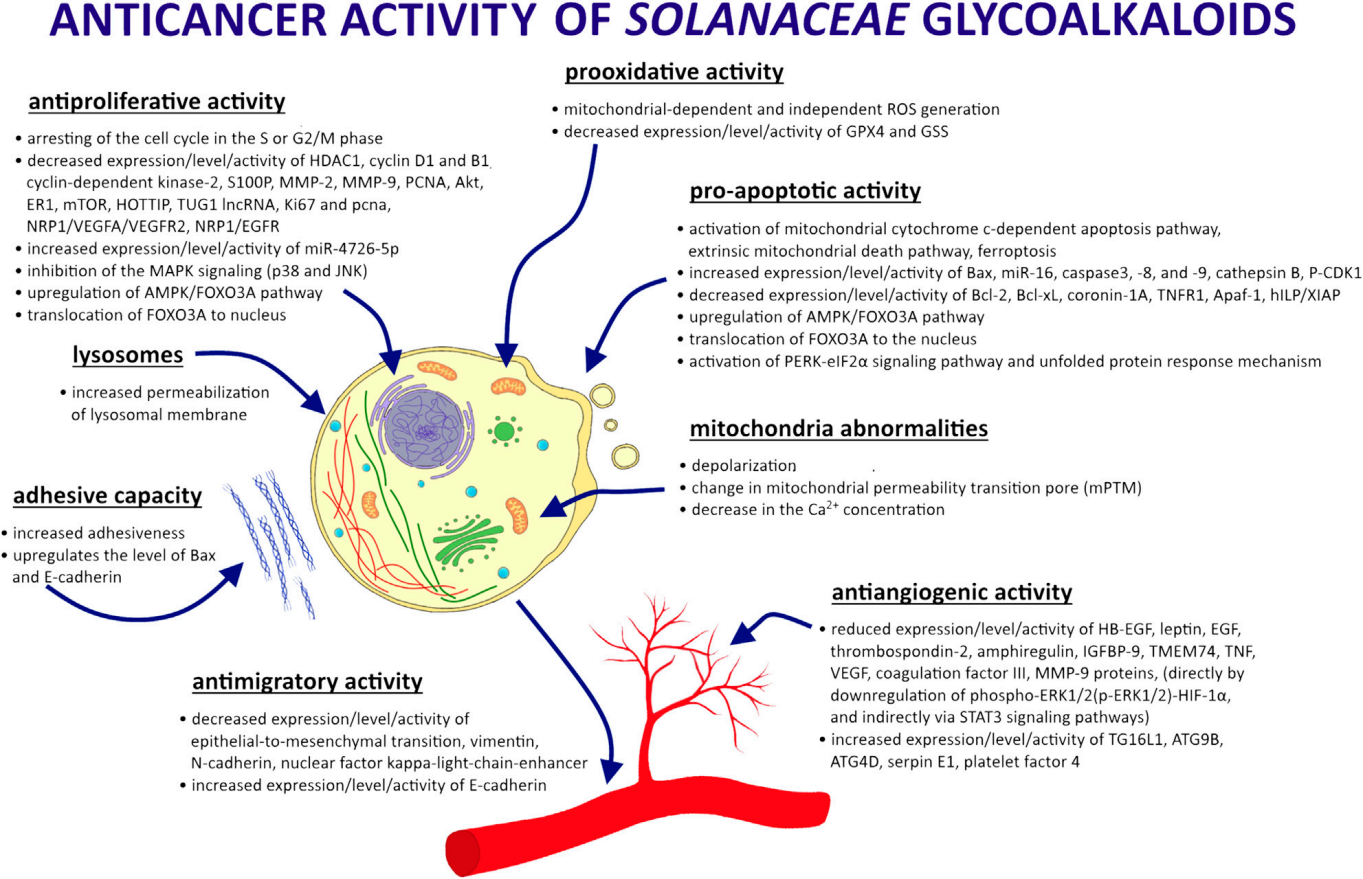
Effect of GA action in cancer cells.

However, GAs from the *Solanaceae* family may have a cytotoxic effect on healthy cells as well as cancer cells. Furthermore, their aglycon parts also have anticancer effects (da Silva et al., 2017; Shen et al., 2017; Akhtar et al., 2018; Gu et al., 2018; Fekry et al., 2019; Ahmad, 2019; Hsieh et al., 2020; Fujimaki et al., 2022; Yu et al., 2020). SN, CH, SS, SM, and TT show antitumor activity against various types of neoplasms. Research results and possible mechanisms of GA activity in cancer cells are shown in Figure 2. Although the mode of action of GAs has been intensively studied, it is not yet fully understood. Both GA extracts from *Solanaceae* plants and compounds used individually have been shown to be effective. The results of recent research on the anticancer properties of *Solanaceae* GAs are presented below, taking into account the different mechanisms of action of these substances (Table 1).

**TABLE 1:** Effect of GAs on cellular targets in cancer cells.

Cancer	Cell line(s)	GA/extract	Type of extract	Dose range [µg/ml]/[mg/kg]	Minimal active concentration/ IC_50_ [µg/ml]	Duration [h]/[day]/[week]	Model	Control	Effect on cellular target	References
ALL	T-ALL Jurkat cells	SN	N/A	0–16 μg/ml	Not stated	24 h	*in vitro*	Negative	Bcl-2↓ Bax ↑	Yi et al. (2018a)
AMCL	THP-1, MV4-11	SS	N/A	0–9 μg/ml	9,893–11051 μg/ml	24–48 h	*in vitro,* mouse and zebrafish xenografts	Negative, positive	AMPK/FOXO3A↑ cyclin B1↓ caspase 9↑ caspase 3↑ Bax↑ P-CDK1↑Bcl-2↓	Zhang H et al. (2020)
AML	AML-193	SN	N/A	0–35 μg/ml	9 μg/ml	24 h	*in vitro*	Negative	Bax↑ miR-16↑ Bcl-2↓	Zheng et al. (2020)
BD	T24, 5637	SS	N/A	4–80 μg/ml	Not stated	24 h	*in vitro*	Negative, positive (DMSO)	NRP1↓ ERK/MAPK↓ P38/MAPK↓ PI3K/AKT↓	Dong et al. (2022)
BC	Bcap-37	SM	N/A	2–9 μg/ml	6 μg/ml	24 h	*in vitro*	Negative	Bax↑ caspase 3↑ Bcl-2↓ Bcl-xL↓	Li et al. (2016)
BC	Bcap-37	SS	N/A	4–18 μg/ml	20 μg/ml	24 h	*in vitro*	Negative	Bax↑ caspase 3↑ Bcl-2↓ Bcl-xL↓	Li et al. (2016)
BC	MCF-7	TT	N/A	103,420–103 μg/ml	Not stated	24–72 h	*in vitro*	Negative, positive (pulmonary fibroblast cell line WI-38)	MMP-2↓ MMP-9↓ MMP-9/NGAL↓	Yelken et al. (2017)
BC	MCF-7	*S. chacoense Bitt.* extract	methanol	50–1,000 mg/ml	Not stated	48 h	*in vitro*	Negative	Bax↑ Bcl-2↓ NFκB↓ CCND1↓ STAT3↓	Cruceriu et al. (2021)
BC	MDA-MB-231	SANT mixture	DMSO	10 μg/ml (solanine)	Not stated	24 h	*in vitro, in vivo* (mouse)	Negative (*in vivo:* normal saline containing 0.5% DMSO)	ATG16L1↑ ATG9B↑ ATG4D↑ TMEM74↓ TNF↓ HB-EGF↓ thrombospondin-2↓ amphiregulin↓ leptin↓ IGFBP-9↓ EGF↓ coagulation factor III↓ MMP-9↓ serpin E1↑ platelet factor 4↑	Li Q et al. (2021)
BC	RT-4	*S. lycocarpum* extract	Not stated	0.625–100 μg/ml	22 μg/ml	48 h	*in vitro,* patient-derived xenograft in mouse	Negative	Bcl-2↓ Bcl-xL↓ survivin↓ PARP↓ Bax↑ caspases↑ MMP-2↓ MMP-9↓	Miranda et al. (2021)
CGC	QBC939	SM	N/A	0–12 μg/ml	Not stated	24 h	*in vitro*	Negative, positive (carbonyl cyanide 3-chlorophenylhydrazone)	Bcl-2↓Bcl extralarge↓ X-linked inhibitor of apoptosis↓ Bax↑ PARP↓ cleaved PARP↑ caspase 3↑ cleaved caspase 3↑ caspase 7↑	Zhang et al. (2018)
CM	CM-319	SM	N/A	0–87 μg/ml	Not stated	48 h	*in vitro*	Negative	DLL1↓ DLL3↓ Notch1↓ HES1↓	Liu Z et al. (2019)
CC	JEG-3	SN	N/A	9–43 μg/ml (*in vitro*), 5 mg/kg/day body weight (*in vivo*)	Not stated	24 h	*in vitro, in vivo* (mouse)	Negative	MMP-2↓ MMP-9↓ PCNA↓ Bcl-2↓ Bax ↑	Gu et al. (2021)
CLC	CT-116, LoVo, SW480, SW620, SW48	TT	N/A	1–12 μg/ml	Not stated	24 h	*in vitro*	Negative	cyclophilin D↑ AIF↑	Rudolf and Rudolf, (2016)
CLC	CT-26	TT	N/A	1–10 μg/ml, 5 mg/kg body wt	Not stated	3–48 h, 2 weeks (*in vivo*)	*in vitro, in vivo*	Negative	survivin↓ AIF↑	Kim et al. (2015)
CRC	SW480, SW620, HT-29	SN	N/A	Not stated	Not stated	Not stated	*in vitro*	Not stated	S100P↓	Ni et al. (2018)
CRC	RKO, HCT-116	SN	N/A	0–22 μg/ml, 5 mg/kg/day (*in vivo*)	Not stated	48 h, 12 days (*in vivo*)	*in vitro, in vivo*	Negative	ROS↑ MMP-2↓ MMP-9↓ caspase3↑ caspase8↑ caspase9↑ cyclinD1↓CDK2↓	Yan et al. (2020)
CRC	HT-29	TT	N/A	0.1–10 μg/ml	Not stated	96 h	*in vitro*	Negative	APC↑	Ishii et al. (2020)
EA	RL95-2	SN	N/A	0–87 μg/ml	23 μg/ml	96 h	*in vitro*	Negative	Akt↓ ER↓ Akt↓ ER↓	Karaboğa Arslan and Yerer, (2018)
EC	EC9706, Eca109	SN	N/A	0–60 μg/ml	Not stated	72 h	*in vitro*	Negative	Bax↑ E-cadherin↑ MMP-2↓ MMP-9↓ Bcl-2↓	Wang L et al. (2016)
EC	EC9706	SN	N/A	0–5 μg/ml, 3.5 mg/kg/day (*in vivo*)	Not stated	48 h, 4 weeks (*in vivo*)	*in vitro, in vivo* (mouse)	Negative, positive (40 μg/ml 5-fluorouracil, 6 μg/ml cisplatin with PBS containing 1‰ DMSO)	miR-138↑ survivin↓	Wu et al. (2018)
EC	EC9706, KYSE30	SN	N/A	0–14 μg/ml	Not stated	24 h	*in vitro*	Negative	miR-138↑ survivin↓	Wang Y et al. (2016)
GC	SGC7901, BGC823	SM	N/A	0–17 μg/ml, 10 mg/kg/day (xenograft)	Not stated	88 h, 18 days (xenograft)	*in vitro,* xenograft in mouse	Negative	Erk1/2 MAPK↓ lncNeat1_2↑	Fu et al. (2019)
GC	SNU1, SNU5	SS	N/A	0–35 μg/ml	Not stated	48 h	*in vitro*	Negative	miR-486-5p/Pi3KR1↑	Zhang H et al. (2020)
GC	SGC-7901	SS	methanol and water	0–50 μg/ml	16 μg/ml	24 h	*in vitro*	Negative	Bcl-2↓ caspase 3↑ Bcl-2/Bax ratio↓	Li et al. (2022)
HCC	HepG2	SN	N/A	2000 μg/ml	Not stated	48 h	*in vitro*	Negative, positive (camptothecin)	ROS↑ ASK1↑ TBP-2↑ JNK↑ p38↑ HDAC1↓	Meng et al. (2016)
HCC	HepG2	SN	N/A	0–10 μg/ml	Not stated	48 h	*in vitro*	Negative	NAT↓ Foxp3↓ TGFβ↓	Gao et al. (2018)
HCC	HepG2	SN	N/A	Not stated	Not stated	Not stated	*in vitro*	Negative	NF-κB↑	Gouhar et al. (2022)
HCC	HepG2	SN	N/A	2–17 μg/ml	Not stated	24 h	*in vitro*	Negative	miR-21↓ MMP-2↓ MMP-9↓	Lin et al. (2020)
HCC	Hep G2	SN	N/A	0–22 μg/ml	Not stated	48 h	*in vitro*	Negative	caspase 3↑ caspase 7↑ Bcl-2↓ survivin↓	El-Daly et al. (2019)
HCC	HepG2	SM	N/A	0–7 μg/ml	Not stated	24 h	*in vitro*	Negative	MMP-2↓ MMP-9↓	Sani et al. (2015)
HCC	HepG2, Huh-7	SM	N/A	0–7 μg/ml, 6 and 30 mg/kg/day (xenograft)	Not stated	72 h, 25 days (xenograft)	*in vitro,* xenograft in mouse	Negative	HOTTIP↓ TUG1↓ miR-4726-5p↑ MUC1↓	Tang et al. (2022)
HCC	HepG2, SMM7721	SM	N/A	4–17 μg/ml	9–11 μg/ml	48 h	*in vitro*	Negative	Ki67↓ Pcna↓ Bcl-2↓ Bax↑ caspase 3↑ caspase 9↑	Xie et al. (2015)
HCC	HepG2	SM	N/A	0–17 μg/ml	Not stated	24 h-2 weeks	*in vitro*	Negative	E-cadherin↑ vimentin↓ N-cadherin↓	Xie et al. (2017)
HCC	HepG2	SS	N/A	0–44 μg/ml, 5–20 mg/kg/day (xenograft)	Not stated	72 h, 15 days (xenograft)	*in vitro,* xenograft in mouse	Negative	miR-375-3p↑ lncRNA CCAT1↓ IRF5↓ SP1↓	Liu J et al. (2019)
HCC	HepG2	SS	Not stated	2–7 μg/ml	Not stated	48 h	*in vitro*	Negative	p53↑ P21↑	Pham et al. (2019)
HCC	HepG2, HepRG	SS	N/A	15–15000 μg/ml, 50 mg/kg body weight (*in vivo*)	Not stated	24 h, 30 days (*in vivo*)	*in vitro, in vivo* (mouse)	Negative	GPX4↓ GSS↓ ROS↑	Jin et al. (2020)
LAC	A549, H1299	SN	N/A	0–13 μg/ml	Not stated	14 days	*in vitro*	Negative	miR-138↑ FAK↓	Zhang et al. (2016)
MG	U87, U251, U118 MG	SS	N/A	0–7 μg/ml	Not stated	14 days	*in vitro, in vivo* (mouse)	Negative	NF-κB↓ P38↓ JNK↓	Wang et al. (2017)
MG	C6	*S. nigrum* extract	methanol	0–0.4 mg/ml, 3.5–14 mg/kg (*in vivo*)	Not stated	48 h, 2 weeks (*in vivo*)	*in vitro, in vivo* (rat)	Negative, positive (Temozolomide)	MMP-2↓ MMP-9↓ Bax↑ cleaved caspase 3↑ Bcl-2↓	Li et al. (2021a)
MB	DAOY	CH	EtOH	1–26 μg/ml	Not stated	24 h	*in vitro*	Negative, positive (Vismodegib)	Smo↓ Hh↓	Gao et al. (2021)
MN	WM239, WM115	SM	N/A	IC50, IC70	Not stated	2 weeks	*in vitro*	Negative, Primary bovine aortic endothelial cells, rat fibroblast, epithelial cells lines	cathepsin B↑ cytochrome c↑ TNFR1↑ hILP/XIAP↓ caspase 3↑ Bcl-xL↑ Bcl-2↑ Bax↓ Apaf-1↓	Al et al. (2016)
MBT	4 T1	SN	N/A	5 mg/kg (*in vivo*)	27–54 μg/ml	72 h, 3 weeks (*in vivo*)	*in vitro, in vivo* (mouse)	Negative, positive (Doxorubicin)	MMP-2↓ MMP-9↓ Bcl-2↓ Bax ↑ mTOR↓ Akt↓	Mohsenikia et al. (2016)
ML	K562/ADM	SN	N/A	2–10 μg/ml	Not stated	24 h	*in vitro*	Negative	MRP1↓	Yi et al. (2018b)
NPC	HNE2, C666-1	SM	N/A	1–12 μg/ml, 3–6 mg/kg (xenograft)	2–3 μg/ml	12 days	*in vitro,* xenograft in mouse	Negative	CCAT1↓ SP1↓ miR7-5p↑	Wu et al. (2020)
NM	SH-SY5Y	TT	N/A	1–26 μg/ml	2 μg/ml	24 h	*in vitro*	Negative, positive (Staurosporine, PMA, Lactacystin, Thapsigargin)	PERK-eIF2α↑	da Silva et al. (2017)
NM	SH-SY5Y	*S. aculeastrum* extract	methanol	2–200 μg/ml	10.72 μg/ml (crude), 17.21 μg/ml (aqueous)	72 h	*in vitro*	Negative	P-glycoprotein↓	Burger et al. (2018)
NSCLC	A549, H1299	SM	N/A	1–7 μg/ml	3–4 μg/ml	72 h	*in vitro*	Negative	Akt↓ SP1↓ NK- κB subunit p65↓ PGE_2_-EP4↓ ERK1/2↑ DNMT1↓ c-Jun↓	Chen et al. (2015)
NSCLC	A549,PC9, H1299,H358, H1650, H1359, H1975	SM	N/A	0–5 μg/ml, 6 mg/kg/day (*in vivo*)	Not stated	72 h, 27 days (*in vivo*)	*in vitro, in vivo* (mouse)	Negative	Stat3↓ IGHBP1↑ FOXO3a↑ SP1↓ PDPK1↓ miR-214-3p↑	Tang et al. (2017)
NSCLC	A549	*S. nigrum* extract	water	20–80 μg/ml, 5–20 mg/kg (*in vivo*)	Not stated	24 h, 15 days (*in vivo*)	*in vitro, in vivo* (mouse)	Negative	FASN-EGFR↓	Shi et al. (2019)
NSCLC	A549	*S. lyratum* extract	Ethanol	0.781–100 μg/ml, 10 g/kg (xenograft in mouse)	Not stated	24 h, 14 days (xenograft in mouse)	*in vitro,* xenograft in mouse	Negative	VEGF↓	Han et al. (2018)
OC	Skov3	TT	N/A	1–3 μg/ml	1–2 μg/ml	72 h	*in vitro*	Negative	PI3K/Akt/mTOR↓ ALDH1↓ Notch1↓ FoxM1↓ c/EBPPβ↓COL11A1↓	Wu et al. (2021)
PCC	-	SN	N/A	300–900 μg/ml	Not stated	24 h	*in vitro*	Negative	VEGF↓ HIF-1α↓ STAT3↓	Wen et al. (2016)
PCC	BxPC-3	SM	N/A	2–8 μg/ml, 4 μg/ml (xenograft)	Not stated	48 h, 21 days (xenograft)	*in vitro,* xenograft in mouse	Negative	Akt/Mtor↓ survivin↓ Ki-67↓ cyclin D1↓ TIMP-2 inhibitor ↑ MMP-2↓	Xie et al. (2019)
PC	DU145	SN	N/A	9–139 μg/ml, 5 mg/kg (xenograft)	28 μg/ml	24 h, 4 weeks (xenograft)	*in vitro,* xenograft in mouse	Negative	ROS↑ p38↑	Pan et al. (2016)
PC	DU145, PC-3	SN	N/A	3–14 μg/ml	Not stated	24 h	*in vitro*	Negative	lncRNA GAS5↑ miR-18a↓	Yang et al. (2019)
PC	PC3, DU145	SM	N/A	1–10 μg/ml, 5 mg/kg/2 days (xenograft)	Not stated	48 h, 4 weeks (xenograft)	*in vitro,* xenograft in mouse	Negative	PI3K/Akt↓	Ge et al. (2022)
PC	CRPC	SM	N/A	1–9 μg/ml, 5–10 mg/kg (xenograft)	6 μg/ml	72 h, 36 days (xenograft)	*in vitro,* xenograft in mouse	Negative	AMPKα↑ MUC1↓ NF-κB subunit p65↓	Xiang et al. (2016)
PC	PC-3	TT	N/A	1–5 μg/ml, 5 mg/kg/3 days (xenograft)	Not stated	72 h, 30 days (xenograft)	*in vitro,* xenograft in mouse	Negative	NF-Κb↓ *Bcl-2↓* phospho-Akt↓ phosphor-ERK1/2↓	Huang et al. (2015b)

ALL, Acute lymphoblastic leukemia; AMCL, acute monocytic leukemia; AML, acute myeloid leukemia; BD, bladder cancer; BC, breast cancer; CGC, cholangiocarcinoma; CM, chordoma; CC, choriocarcinoma; CLC, colon cancer; CRC, colorectal cancer; EA, endometrial adenoma; EC, esophageal cancer; GC, gastric cancer; HCC, hepatocellular carcinoma; LAC, lung adenocarcinoma; MG, malignant glioma; MB, medulloblastoma; MN, melanoma; MBT, metastatic breast tumor; ML, myelogenous leukemia; NPC, nasopharyngeal carcinoma; NM, neuroblastoma; NSCLC, non-small cell lung cancer; OC, ovarian cancer; PCC, pancreatic cancer; PC, prostate cancer. N/A—not applicable.

## Antitumor effects of *Solanaceae* glycoalkaloids

### Solanine

Solanine (SN) is one of the two principal *S. tuberosum* L. GAs, in addition to chaconine (CH), but it also occurs in many other *Solanum* species. The SN level in potatoes depends on many factors, such as light and temperature. SN is composed of trissacharide solatriose (D-glucose, D-galactose, ʟ-rhamnose) attached to the aglycon part solanidine (Milner et al., 2011). SN exhibits fungicidal (Boulogne et al., 2012), antibacterial (Rajalakshmi and Jayachitra, 2017) and anti-inflammatory activity depending on inhibition of the NF-κB signaling pathway (Shin et al., 2016). SN also exhibits an antitumor effect on different cancer cells.

Most studies of SN anticancer activity were conducted in HepG2 cells of hepatocellular carcinoma (HCC). SN promotes the production of ROS by human HCC HepG2 cells in both mitochondrial-dependent and mitochondrial-independent manners. This compound also upregulates the expression and kinase activity of ASK1 and TBP-2, which are proteins that activate the JNK and p38 signaling pathways. Furthermore, SN decreases the expression level of the proliferation-associated protein HDAC1. Thus, it contributes to cell apoptosis (Meng et al., 2016). Arylamine N-acetyltransferase (NAT) is an enzyme involved in the transformation of arylamines into carcinogens. SN inhibits NAT activity and downregulates its expression in HCC cells (Gao et al., 2018).

The presence of CD4^+^CD25^+^ Treg cells in peripheral blood and lymph nodes could be a marker of many types of cancer. The indicator of these cells is a Foxp3 forkhead transcription factor that is involved in the proper functioning of Treg cells. TGFβ is a growth factor of epithelial-derived cells that suppresses the response of the immune system. SN promoted the antitumor immune response by decreasing the proportion of CD4^+^CD25^+^ Treg cells and negatively impacting the expression of Foxp3 and TGFβ in mouse HCC cells. The tumor size in the transplanted tumor model in mice was smaller than that in the control (Gao et al., 2020). SN generates oxidative stress in HCC HepG2 cells by upregulation or downregulation of appropriate miRNAs controlling the NF-κB pathway and increasing NF-κB expression (Gouhar et al., 2022, p. 202). SN inhibits the proliferation and migration induced by acetylcholine in HCC Hep G2 cells by attenuating the epithelial-mesenchymal transition. It reduces the activity of matrix metalloproteinases, which play a key role in the development of the neoplastic process and the formation of metastases in tumors of various origins. SN had an inhibitory effect on extrahepatic metastasis by lowering microRNA-21 expression in exosome-treated A549 lung cancer cells (LC) (Lin et al., 2020).

Studies on AML-193 acute myeloid leukemia (AML) cells show that SN causes morphological changes in neoplastic cells and has a dose-dependent pro-apoptotic effect. SN changes the expression of genes that encode the Bax and Bcl-2 proteins, which are markers of apoptosis. SN increases the expression of Bax and miR-16 (noncoding RNA genes that regulate gene expression), resulting in a decrease in Bcl-2 expression, thus inhibiting cell proliferation (Zheng et al., 2020). SN has cytotoxic effects on colorectal cancer (CRC) *in vitro* and *in vivo*. It inhibits the growth, migration, and invasion of CRC SW480, SW620, and HT-29 cells and promotes cell cycle arrest as well as apoptosis. Additionally, SN decreases the expression of S100P, which is involved in the regulation of many cellular processes, e.g., cell cycle progression and differentiation. *In vivo*, this GA inhibits tumor growth (Ni et al., 2018). Akt serine-threonine kinase is the main effector of phosphatidylinositol 3-kinase PI3K, contributing to cell survival by mediating its response to the action of growth factors. Disturbances in the functioning of this transmitter are observed in many disorders, e.g., cancer. SN reduces the expression of genes and the activity of Akt and ER proteins in RL95-2 endometrial adenoma cells (EA) containing the ER estrogen receptor. This may result in inhibition of the PI3K/Akt and ER signaling pathways. The single-administered GA effect was as strong as that of the Akt API-1 inhibitor and the ER-MPP blocker (Karaboğa Arslan and Yerer, 2018). SN inhibits the growth, proliferation, migration, and invasion and promotes apoptosis of human esophageal EC9706 and Eca109 cancer cells (EC) *in vitro*. It upregulates the levels of Bax and E-cadherin, which regulate the adhesive capacity of cells. However, SN downregulates the expression of MMP-2, MMP-9, and Bcl-2 (Wang L. et al., 2016). SN induces apoptosis in human choriocarcinoma (CC) JEG-3 cells. Moreover, it inhibited proliferation, invasion, and migration *in vitro* and *in vivo* in mice. SN decreases MMP-2, MMP-9, proliferative cellular nuclear antigen (PCNA), and Bcl-2 levels, while it increases Bax expression (Gu et al., 2021). SN regulates the expression of cell cycle proteins in prostate cancer (PC) DU145 cells. In addition, it induces apoptosis through the ROS and P38 MAPK kinase signaling pathways. SN also reduces tumor cells *in vitro* and *in vivo* (Pan et al., 2016). Under hypoxic conditions, increased expression of crucial regulators of the angiogenesis process was found, such as hypoxia-inducible factor-1 nuclear transcription factor, hypoxia inducible factor-1α (HIF-1α) and phosphorylated signal transducer and activator of transcription 3 (p-STAT3). VEGF is a proangiogenic growth factor that stimulates the formation of new blood vessels, while inhibition of tumor angiogenesis is crucial because it leads to growth inhibition and reduced invasiveness and metastasis formation. SN reduces the expression of VEGF directly by downregulating phospho-ERK1/2(p-ERK1/2)-HIF-1α and indirectly *via* STAT3 signaling pathways in pancreatic cancer (PCC) cells. Thus, downregulation of VEGF is a combined result of HIF-1α and STAT3 action (Wen et al., 2016). SN exhibited cytotoxic effects in human CRC RKO and HCT-116 cells *in vitro* and *in vivo*. It inhibits their proliferation, migration, invasion, and adhesion and induces apoptosis as a result of increased ROS generation and caspase 3, 8, and 9 activation. SN treatment leads to cell cycle arrest in the G0-G1 phase. It decreases the expression levels of cyclin D1 and cyclin-dependent kinase 2. Additionally, SN reduces the expression level and activity of MMP-2 and MMP-9 (Yan et al., 2020). SN showed an even greater cytotoxic effect on human breast cancer (BC) MCF-7 and MDA-MB-231 cells than cycloheximide, which was used as a positive control. Furthermore, SN has an apoptosis-inducing potential and arrests MCF-7 cells in the S phase (Akter et al., 2015). In *in silico* studies, *S. nigrum* L. GAs have been identified that may bind to selected cytoskeleton proteins. SN has a binding affinity for coronin-1A, which is a protein that inhibits intrinsic pathway-mediated apoptosis; therefore, inhibiting its function could potentially be a target for anticancer therapy (Ahmad, 2019). SN distributed *via* biocompatible polymeric carriers called dendrosomes (DNS) to metastatic breast tumor (MBT) 4T1 cells was more efficient in treatment *in vitro* and *in vivo* and safer than SN alone. DNS decreased the proliferation of cells and increased the expression of Bax while causing lower expression levels of Bcl-2, MMP-2, MMP-9, mTOR, and Akt (Mohsenikia et al., 2016).

SN also exhibits potential for use in combination with other pharmaceuticals, such as fluorouracil and cisplatin. Fluorouracil is an analog of pyrimidine, which blocks thymidylate synthetase, disrupts DNA synthesis, and blocks the cell cycle in the S phase. Cisplatin is an alkylating agent that attaches alkyl groups to DNA bases, leading to fragmentation of DNA. SN increased the sensitivity of EC9706 and KYSE30 EC cells to these chemotherapeutic agents (Wu et al., 2018). MiR-138 is a microRNA that targets genes associated with proliferation, migration, invasion, and apoptosis. Survivin is a protein involved in regulation of the cell cycle and inhibition of apoptosis. SN induces apoptosis by increasing miR-138 and reducing the expression of the survivin gene. SN also enhanced the action of these cytostatics in EC9706 cells transplanted into mice. MiR-138 inhibitors reverse the effect induced by SN, and overexpression of the gene encoding survivin protects tumor cells from apoptosis (Wu et al., 2018). SN also amplifies the anticancer effect induced by cisplatin in HepG2 HCC cells, such as growth inhibition and induction of apoptosis. The mechanism of action is to stop the cell cycle in the G2/M phase, stimulate DNA fragmentation, and induce cell death by activating caspases 3 and 7. SN also reduces the expression of genes encoding the antiapoptotic proteins Bcl-2 and surviving, and regulates the expression of microRNA-21 (El-Daly et al., 2019). SN also increases the radiosensitivity and chemosensitivity of human lung adenocarcinoma (LAC) A549 and H1299 cancer cell lines by upregulating miR-138 and downregulating focal adhesion kinase (FAK) mRNA and protein expression (Zhang et al., 2016). SN induces apoptosis of acute lymphoblastic leukemia (ALL) human T-ALL Jurkat cells. It increases the level of Bax and reduces Bcl-2 mRNA and protein levels. Furthermore, SN increases the chemosensitivity of these cells to adriamycin (Yi et al., 2018a) and reverses the multidrug resistance of human myelogenous leukemia (ML) K562/ADM cells (Yi et al., 2018b). Treatment with this compound reduces cell proliferation, leads to higher concentrations of adriamycin in cells, and reduces the expression of the MRP1 protein dependent on the JNK pathway (Yi et al., 2018b). SN also increases the radiosensitivity of human PC and EC cells. This compound causes upregulation of the lncRNA GAS5, leading to negative regulation of miR-18a expression by target binding in PC cells (Yang et al., 2019). SN upregulates miR-138 expression in EC cells, leading to decreased expression of survivin genes and increased radiotherapy efficacy (Wang Y. et al., 2016).

### Chaconine

Chaconine (CH) was identified in *S. tuberosum* L. in 1954. The SN and CH ratios in potatoes can differ because they vary, e.g., between cultivars. Chaconine contains solanidine as an aglycone unit and a chacotriose carbohydrate chain (D-galactose and two moieties of ʟ-rhamnose) (Milner et al., 2011). This GA is a bioactive compound with fungicidal activity (Boulogne et al., 2012). Furthermore, CH has been shown to have anti-inflammatory properties *in vitro* and *in vivo* and potency for use in the treatment of sepsis (Lee et al., 2015).

There are only a few recent studies on the anticancer effects of CH. Its role in the inhibition of Hh-dependent tumors, such as medulloblastoma (MB), has been determined. This compound reduces Hh pathway activity by binding to the Smo protein. Furthermore, CH exhibits an antiproliferation effect and decreases the growth of MB DAOY cells. The viability of cells treated with CH was even lower than that of cells treated with 22-NHC hydrochloride, a new-generation Smo inhibitor (Gao et al., 2021). CH also decreases the expression of the genes encoding Akt (serine-threonine protein kinase) and ERα (estrogen receptor α) and their activity in the RL95-2 estrogen receptor-positive human endometrial cancer cell line. Thus, it seems that CH may exert suppressive effects on the PI3K/Akt and ERα signaling pathways (Karaboğa Arslan and Yerer, 2018).

### Solamargine

Solamargine (SM) can be found in the aubergine *Solanum melongena* L., but it was also isolated from other *Solanaceae* species. Solasodine is its hydrophobic unit, and it has the same carbohydrate chain as SN (D-glucose, D-galactose, and ʟ-rhamnose) (Milner et al., 2011). In addition to anticancer activity, SM also exhibits insecticidal and fungicidal effects (Boulogne et al., 2012).

SM is the most active compound of the GAs isolated from the fruit of *S. melongena L.* It shows cytotoxicity against HCC Huh7 and HepG2 liver cancer cells. The antiproliferative properties of this GA are due to its ability to arrest the cell cycle in the S phase. This compound shows significant pro-apoptotic properties in both of the above cancer cell lines (Fekry et al., 2019). SM significantly reduces HepG2 HCC cell migration and invasion *in vitro* by decreasing the expression levels of the MMP-2 and MMP-9 genes and reducing the activity of related proteins (Sani et al., 2015). SM also acts through the HOTTIP-TUG1/miR-4726-5p/MUC1 signaling pathway, which inhibits the growth of HCC HepG2 and Huh-7 cells *in vitro* and *in vivo*. The compound downregulates the expression levels of HOTTIP and TUG1 lncRNA and upregulates the expression of miR-4726-5p, which inhibits the expression of the MUC1 protein. Furthermore, this GA enhances the antitumor effect of sorafenib, a drug used in HCC treatment (Tang et al., 2022). SM exhibits an antitumor effect in HHC cells, SMM7721 cells, and HepG2 cells. It inhibits proliferation and induces apoptosis and cell cycle arrest in the G2/M phase. SM also reduces the levels of proteins involved in proliferation (Ki67 and pcna) and the antiapoptotic protein Bcl-2. Moreover, it increases the activity of the pro-apoptotic proteins Bax, caspase 3 and caspase 9 (Xie et al., 2015) and suppresses the migration and invasion of HCC HepG2 cells through the inhibition of epithelial-to-mesenchymal transition (EMT), which is necessary for tumor growth. SM upregulates the expression of the EMT-associated epithelial marker E-cadherin and downregulates the expression of the mesenchymal markers vimentin and N-cadherin (Xie et al., 2017).

SM exhibits cytotoxic effects on human BC Bcap-37 cells; it caused apoptosis and depolarization of mitochondria after treatment for 24 h. SM increases the expression levels of Bax and caspase 3 and downregulates the expression levels of Bcl-2 and Bcl-xL. SM induces the mitochondrial cytochrome c-dependent apoptosis pathway (Li et al., 2016). SM showed an even greater cytotoxic effect on human BC MCF-7 and MDA-MB-231 cells than cycloheximide, which was used as a positive control (Akter et al., 2015). SM exhibits cytotoxic activity against breast adenocarcinoma (BAC) MDA-MB-231, LC A549, HCC Hep3B, and PC PC3 cancer cell lines (Tai et al., 2018). Research on mouse melanoma (MN) model B16F10 cells discovered that SM inhibits proliferation and decreases tumor size (Furtado et al., 2022). This glycoalkaloid also suppresses the growth of MN WM239 and WM115 cells. It promotes permeabilization of the lysosomal membrane. SM increases the level of cathepsin B, which contributes to the release of cytochrome c and upregulation of TNFR1, leading to the extrinsic mitochondrial death pathway. In addition, SM downregulates hILP/XIAP, which contributes to caspase 3 cleavage, upregulation of Bcl-xL and Bcl2, and downregulation of Apaf-1 and Bax proteins in cells. Thus, the classic intrinsic apoptosis pathway is probably not involved in the mechanisms of action of this compound (Al et al., 2016). SM suppresses the growth of human non-small cell lung cancer (NSCLC) by influencing the phosphatidylinositol 3-kinase/Akt (PI3-K/Akt) signaling pathway *in vitro* and *in vivo*. Prostaglandin E2 (PGE_2_) belongs to the protein family and is a ligand for G-coupled receptors involved in processes such as growth and metastasis. Inhibition of Akt phosphorylation by GA causes decreased expression of SP1 and NK-κB subunit p65 transcription factors, which contribute to inhibition of the expression of the PGE_2_ E-prostanoid receptor 4 (EP4) protein (Chen et al., 2015). Subsequently, it was found that decreased EP4 expression results in enhanced ERK1/2 signaling, leading to downregulation of DNA methyltransferase 1 (DNMT1) and c-Jun transcription factor expression; DNMT1 is an enzyme that regulates cell survival, cell cycle arrest, and cell death, and c-Jun is a subunit of activating protein-1 (AP-1) that activates oncogenes (Chen et al., 2017). Moreover, SM inactivates Stat3, causing upregulated expression of the IGHBP1 gene that leads to correlative modulation of FOXO3a (+) and SP1 (-). These interactions caused decreased growth and induce cell cycle arrest in NSCLC cells *in vitro* and *in vivo*. Additionally, SM enhances the anticancer effect caused by metformin (Tang et al., 2017). SM inhibited NSCLC cell growth *in vitro* and *in vivo* by regulating the interaction between antisense RNA lncRNA HOX transcript (HOTAIR) and miR-214-3p. It results in a downregulation of the expression of the 3-phosphoinositide-dependent protein kinase-1 (PDPK1) gene (Tang et al., 2019).

SM suppresses gastric cancer (GC) progression by decreasing the phosphorylation of extracellular signal-regulated kinase (Erk) 1/2 mitogen-activated protein kinase (MAPK). In this way, the compound induces the expression of the long noncoding RNA nuclear paraspeckle assembly transcript 1 (lncNeat1_2). Furthermore, SM inhibits GC growth *in vivo* (Fu et al., 2019). SM downregulates the PI3K/Akt pathway and suppresses PC growth *in vitro* and *in vivo*. This signaling pathway seems to be responsible for the chemoresistance of this disease. Furthermore, it increases the anticancer effect caused by docetaxel; thus, it may have the potential to be used in therapy together with anticancer drugs (Ge et al., 2022). SM activates AMPKα, reducing the protein expression of MUC1 and NF-κB subunit p65 *in vitro* in castration-resistant PC CRPC cells and inhibiting tumor growth *in vivo*. Furthermore, it acts synergistically with metformin (Xiang et al., 2016). SM also shows an antitumor effect in CM-319 human chordoma (CM) cells. It causes cell cycle arrest in the G1 phase, inhibiting proliferation and inducing apoptosis. SM also significantly reduced the expression levels of DLL1, DLL3, Notch1, and HES1, modulating the Notch pathway (Liu J. et al., 2019). SM inhibits the growth of nasopharyngeal carcinoma (NPC) cells *in vitro* and *in vivo* by modulating the expression of colon cancer-associated transcript-1 (CCAT1) of lncRNA and miR7-5p. It downregulates CCAT1 and SP1 protein expression and upregulates miR7-5p expression levels (Wu et al., 2020, p. 201). SM induces apoptosis, arrests the cell cycle, inhibits PCC metastasis *in vitro,* and suppresses PCC growth *in vivo*. SM acts through inhibition of the Akt/mTOR signaling pathway. The compound downregulates the expression of survivin, Ki-67, and cyclin D1. SM also decreases MMP-2 and increases the expression of metallopeptidase inhibitor-2 of TIMP (Xie et al., 2019). PCC treatment with SM delivered to cells in Fe_3_O_4_ magnetic liposomes (MLP) enabled a slow release of the GA and was more effective than SM applied alone. For example, MLPs-SM inhibited Ki-67 expression and caused increased apoptosis compared to SM (Xie et al., 2019). SM induces apoptosis of human cholangiocarcinoma (CGC) QBC939 cells through the mitochondrial pathway and changes the mitochondrial membrane potential *in vitro*. This compound downregulates the gene expression of Bcl-2, an extralarge and X-linked inhibitor of apoptosis, but upregulates the expression of the Bax gene. Furthermore, SM decreases the level of PARP proteins while increasing the levels of cleaved PARP, caspase 3, cleaved caspase 3 and caspase 7 (Zhang et al., 2018).

Cancer stem cells (CSCs) seem to be the main factors responsible for cancer drug resistance. *In silico* studies have shown that SM can target CSCs by modifying the sonic hedgehog (SHH) pathway. It also has good binding ability to the Gli protein, which is a transcription factor that mediates transcription related to cancer and CSCs (Mayank, 2016). Furthermore, SM was used as a component in the optimization of new nanoparticles with a bioresponsive off-coating property and an active tumor-targeting ability. These particles have longer circulation, increased accumulation, and penetration efficacy in the cancer area (Li et al., 2021a).

### Solasonine

Solasonine (SS) has the same aglycon part as SM (solasodine), and its carbohydrate chain is identical to that of SN (solatriose). It occurs in many *Solanum* species, such as *S. melongena* L, *S. berthaultii* Hawkes*, S. platanifolium* Sims*,* and *S. ambosinum* Ochoa (Milner et al., 2011). SS has insecticidal activity, e.g., against *G. mellonella* larvae (Spochacz et al., 2021).

Similar to SM, SS can target CSCs by modifying the Hh pathway (Mayank, 2016). The SHH (sonic hedgehog) signaling pathway plays an important role in the growth of neoplastic tumors and in maintaining their resistance to treatment. Stimulation of the SHH pathway increases the expression of genes encoding Patched (PTCH1) and Smoothened (SMO) proteins. PTCH1 binds to the SHH protein, and SMO is responsible for activation of the signaling cascade leading to translocation of GLI transcription factors into the cell nucleus and activation of the transcription of SHH-dependent genes such as HOX, WNT, FGF-4, VEGF, CAPN1, and NRP, causing cell proliferation (Yang et al., 2016). One of the mechanisms of resistance to treatment is the presence of Smo proteins; therefore, their inhibitors are being tested. Research indicates the possibility of using SS in anticancer therapies because it reduces the resistance of cancer cells to the currently used SMO inhibitors. SS significantly inhibits the activity of the SHH pathway as a result of its interaction with the transcription factor Gli. SS inhibits ALP (alkaline phosphatase) in murine C3H10T1/2 mesenchymal cells and reduces the expression of the *Gli1* and *Ptch1* genes but does not affect the activity of transcription factors induced by TNF-α (tumor necrosis factor α) cytokines and PGE2 prostaglandin, indicating the selective action of SS on cells (Yang et al., 2016).

SS may inhibit the growth of HCC cells through regulatory interactions between miR-375-3p, lncRNA CCAT1, transcription factor SP1, and interferon regulatory factor IRF5, as demonstrated *in vivo* in a mouse model. This GA increases miR-375-3p expression and decreases levels of lncRNA CCAT1, and consequently leads to SP1-mediated reduction of IRF5 expression, which participates in the regulation of the expression of genes involved in the immune response (Liu J. et al., 2019). Mortalin is a protein that is overexpressed in cancer cells by blocking the action of the transcription factor p53. The arrest of this signaling pathway activates the apoptosis process in HepG2 HCC cells, which has been confirmed *in vitro*. *In silico* studies have shown that SS is a potential inhibitor of the p53-mortalin interaction. Furthermore, SS-induced cell death has been shown to occur in cells that do not express the p53 protein, indicating that this process can also be regulated by p53-independent signaling pathways (Pham et al., 2019). Often, the target of anticancer therapies is microtubules (Jordan and Wilson, 2004). *In silico* studies show the highest binding affinity for coronin-1A of SS among all identified *S. nigrum* L. GAs, higher than SN (Ahmad, 2019). SS isolated from *S. melongena* L. fruit shows cytotoxicity against HCC Huh7 and HepG2 liver cancer cells. The antiproliferative properties of this GA are due to its ability to arrest the cell cycle in the S phase. The compound has a significant pro-apoptotic effect on Huh7 cells. (Fekry et al., 2019). SS from *S. nigrum* L. fruits also exhibited cytotoxic effects in MGC803, HepG2 and SW480 cancer cell lines, however, the LD_50_ values were higher than that of the positive control KPT330 (Gu et al., 2018). The marker of ferroptosis, which is a form of cell death, is an iron-dependent accumulation of lipid peroxides. In the protection mechanism against this process, glutathione peroxidase 4 (GPX4) is involved, and converts lipid peroxides into nontoxic lipid alcohols. To create a reduced form of GSH, which is the cofactor of GPX4, glutathione synthetase GSS is involved; SS inhibits the expression of GPX4 and GSS, increasing ROS levels and inducing ferroptosis in HepG2 and HepRG cells. The results of research indicate that this GA inhibits proliferation both *in vivo* and *in vitro*. SS arrests the cell cycle in the G2/M phase and suppresses the migration and invasion of HCC cells (Jin et al., 2020).

In addition, SS inhibits the proliferation and migration of malignant glioma (MG) human U87 MG, U251 MG and U118 MG cells by reducing the activity of the nuclear factor kappa-light-chain-enhancer of activated B cells (NF-κB) signaling pathway. SS also inhibits the signaling of mitogen-activated protein kinases (MAPKs), namely, p38 and JNK (c-Jun N-terminal kinases). MAPKs are protein kinases that regulate the response to external signals reaching the cell (mitogens) and therefore have an impact on gene expression, division, differentiation, migration, and cell apoptosis (Wang et al., 2017). The latest research shows that SS suppresses neuropilin-1 NRP1 expression in bladder cancer (BD) cells by binding to the b1 domain of the protein on the cell membrane. The NRP1/VEGFA/VEGFR2 and NRP1/EGFR complexes cannot be created, which leads to inhibition of the ERK/MAPK, P38/MAPK, and PI3K/AKT signaling pathways. Furthermore, NRP1 is retained in the cytoplasm, facilitating its degradation. Therefore, this GA probably acts in two ways: intra- and extracellularly (Dong et al., 2022). SS exhibited cytotoxic effects in human BC Bcap-37 cells, and caused apoptosis and depolarization of mitochondria after treatment for 24 h. SS increases the expression levels of Bax and caspase 3 and downregulates the expression levels of Bcl-2 and Bcl-xL. SS induces the mitochondrial cytochrome c-dependent apoptosis pathway (Li et al., 2016). SS showed an even greater cytotoxic effect on BC MCF-7 and MDA-MB-231 cells than cycloheximide, which was used as a positive control (Akter et al., 2015). SS also has anticancer properties against GC; it inhibits its growth and progression, and the mechanism of apoptosis depends on the upregulation of the miR-486-5p/Pi3KR1 pathway (Zhang H. et al., 2020). SS shows cytotoxicity on human GC SGC-7901 cells in a dose-dependent manner. It suppresses proliferation to a degree comparable to that of cisplatin and arrests the cell cycle, mainly in the G2 phase. This compound downregulates the Bcl-2 protein, which controls the mitochondrial permeability transition pore (mPTM), causing a decrease in the Ca^2+^ concentration within the mitochondria, which leads to an increase in the amount of Ca^2+^ ions in the cell and a reduction in the mitochondrial membrane potential. Therefore, caspase 3 activation and reduction of the Bcl-2/Bax ratio occur, which contribute to cell cycle arrest in phase M. Therefore, SS is believed to induce the mitochondrial apoptotic pathway because, in addition to Bcl-2, it also decreases the level of Bax expression (Li et al., 2022). SS shows antitumor activity against the acute monocytic leukemia (AMCL) cell lines THP-1 and MV4-11. SS upregulates the AMPK/FOXO3A pathway, inducing FOXO3A translocation to the nucleus. It leads to the induction of cell cycle arrest in the G2/M phase because it inhibits the expression of cyclin B1 and apoptosis as a result of promoting the expression of caspase 9 and caspase 3. SS increases Bax and P-CDK1 expression and downregulates Bcl-2 expression. In addition, it inhibited tumor growth *in vivo* in mice and zebrafish xenograft models (Zhang Y. et al., 2020).

Sevofluran is an inhalant anesthetic that shows adverse effects on the central nervous system, such as neuronal apoptosis and neuroinflammation. SS has the ability to reduce damage in the mouse hippocampal neuron cell line HT22 caused by the anesthetic by activating the AMPK/FoxO3a pathway. Moreover, AMPK inhibition greatly influences the inhibitory effect of SS on apoptosis, oxidative stress and inflammation. Furthermore, this GA decreased sevoflurane-induced learning and memory disorders *in vivo* in mice (Zhang and Yan, 2022). Comparison of these two studies shows that the mechanisms of apoptosis in AMCL and neuronal cells are different because the upregulation of the AMPK pathway can lead to apoptosis or, in contrast, to apoptosis inhibition.

### Tomatine

Tomatine (TT) is a steroidal glycoalkaloid that occurs in large amounts in the leaves of tomatoes (*S. lycopersicum* L.). It is composed of an aglycon, a hydrophobic part called tomatidine, a hydrophilic carbohydrate chain consisting of two glucose molecules, one galactose and one xylose molecule (lycotetraose), and a polar amine group. TT is a biologically active substance and, in addition to its anticancer properties, has a wide range of activities, such as insecticidal (Ventrella et al., 2016), fungicidal (Boulogne et al., 2012), antibiotic (Friedman, 2002) and anti-inflammatory (Zhao et al., 2015) effects.

TT at a concentration of 10 μg/ml inhibited CRC HT-29 cell proliferation 24 h after administration but did not induce apoptosis. It increases the expression of the gene encoding the APC protein that is involved in the regulation of the cell cycle (Ishii et al., 2020). Other studies indicate that TT shows pro-apoptotic properties against metastatic melanoma (MSMN) cells. This GA also reduces cell invasion and inhibits angiogenesis, possibly by affecting the ER/p-eIF2/VEGF pathway (Serratì et al., 2020). TT inhibits the growth and apoptosis of human myeloid leukemia (MLL) HL-60 cells in a dose-dependent manner. The cytotoxic effect is based on its ability to bind to cholesterol and, consequently, disrupt the cell membrane and lead to necrosis. TT also inhibited the growth of xenografts of these cancer cells in mice (Huang et al., 2015a). This GA acts through a caspase-independent mechanism (Del Giudice et al., 2015). This was confirmed by another study on the SH-SY5Y neuroblastoma (NM) cell line. TT- and tomatidine-induced cell death has been demonstrated to be independent of caspases and receptor-interacting protein 1 (RIP1) threonine kinase because exposure to the pan Z-VAD-caspase inhibitor fmk and necrostatin-1 (an inhibitor of RIP1 kinase) did not prevent this process. Furthermore, both compounds increase the level of calcium ions in the cell. The increasing number of misfolded proteins in the endoplasmic reticulum activates the UPR (unfolded protein response) stress response mechanism and the activation of receptors: PERK (protein kinase RNA-like endoplasmic reticulum kinase), IRE1 (inositol-requiring-enzyme 1) and ATF6 (activating transcription Factor 6). As a result of the action of TT and tomatidine, the PERK-eIF2α signaling pathway is activated, but the IRE1α pathway is not. Furthermore, TT is an inhibitor of the 20S proteasome, so it influences the concentration of proteins in the cell (da Silva et al., 2017). The increased level of ROS causes overexpression of the gene encoding mitogen-activated protein kinase 6 (MAPK6), which leads to the creation of neoplasms. *In silico* molecular docking experiments have shown that TT may suppress the expression of MAPK6/ERK3 proteins and protect cells against cancer induced by oxidative stress (Kalimuthu et al., 2021).

TT suppresses proliferation and inhibits autophagy to induce apoptosis in Skov3 cells of ovarian cancer (OC), which was confirmed by measuring the expression of the *Beclin-1* gene. Additionally, this GA inhibits the PI3K/Akt/mTOR pathway (Wu et al., 2021). TT induces necroptosis and caspase-independent apoptosis in human colon cancer (CLC) cells, especially those with a hyperactivated PI3K/AKT pathway. This GA changes the expression of genes that encode the RIP3 and RIP1 proteins and causes the accumulation of mitochondrial cyclophilin D. The effects of TT treatment include lysosomal membrane permeabilization and mitochondrial secretion of apoptosis-inducing factor (AIF), which lead to cell death probably through the JNK pathway (Rudolf and Rudolf, 2016). TT induces the death of CLC CT-26 cells through caspase-independent pathways *in vitro* and *in vivo*. It promotes the nuclear translocation of AIF and downregulates survivin gene expression. TT generated approximately 50% lysis of cancer cells 24 h after application at a concentration of 3.5 µM (Kim et al., 2015). Matrix metalloproteinases (MMPs) secreted from tumor cells are key factors in neoplasm metastasis. Research shows that TT increased apoptosis and suppressed the activation of MMP-2, MMP-9 and MMP-9/NGAL in the human BC cell line MCF-7 treated with this compound (Yelken et al., 2017).

TT may also have the potential to be used in combined therapy. Porphyrin complexes of platinum are used in photodynamic therapy, where singlet oxygen is formed under the influence of light, which results in the death of tumor cells. Because of its chemical properties, TT creates self-assembled nanostructured supramolecules. Therefore, it could be used in combined therapy with Pt complexes as it improves their incorporation into cells and increases the efficiency of photodynamic therapy (Fujitsuka et al., 2021). In addition, TT and curcumin used in combination inhibited growth more effectively and promoted apoptosis in PC-3 cells *in vitro.* These substances synergistically suppress the activity of NF-κB and decrease the expression of *Bcl-2*. The TT-curcumin effect was also observed as decreased levels of phospho-Akt and phospho-ERK1/2 in cancer cells. This combined therapy also slowed cancer cell growth *in vivo* (xenografts in mice) (Huang et al., 2015b).

### Crude extracts of *Solanaceae* plants

GA extracts of *Solanaceae* plants have also been studied due to their anticancer properties. The ripe fruit extract of *S. nigrum* L. shows potent antitumor properties *in vitro* (LC A549 cells) and *in vivo* in mice with Lewis tumors in a dose-dependent manner. Cancer cells show increased expression of genes encoding fatty acid synthase (FASN), which correlates with resistance to anticancer drugs targeting the epidermal growth factor receptor (EGFR). Both *S. nigrum* L. extract and the newly identified GA of this plant, solaoiacid, inhibit the FASN-EGFR signaling pathway and affect the migration, autophagy, apoptosis, and immune response of non-small cell lung cancer (NSCLC) cells. Furthermore, solaoiacid acts with an IC_50_ = 2.3 μmol/L, which is significantly lower than that of SM (Shi et al., 2019). The extract of *S. nigrum* L. had antitumor effects on rat C6 MG *in vitro* and *in vivo*. It inhibits the viability, proliferation, migration, and invasion of cancer cells. Moreover, the extract induced annexin V + PI + late-stage apoptosis and decreased the protein levels of MMP-2 and MMP-9. It also upregulates Bax and cleaved caspase 3 and downregulates the expression of Bcl-2. In rat brains, the extract of *S. nigrum* L. inhibited the proliferation, growth, and penetration of cancer tissue (Li et al., 2021a). The methanolic extracts of the dried root, stem, and leaves of *S. nigrum* L. diminished proliferation and promoted apoptosis in the human cancer cell lines MDA and A549 and, particularly, HepG2. Healthy Vero cells were significantly vulnerable to the extract. Moreover, molecular docking analysis shows *S. nigrum* L. GA binding affinity to vimentin, which is thought to be a marker of epithelial-mesenchymal transition and is crucial for cancer metastasis (Ahmad et al., 2017). Vimentin belongs to the group of intermediate filament (IF) proteins, which are one of the three systems of the cell cytoskeleton, in addition to actin and myosin filaments and microtubules. IFs are highly flexible and have an α-helical conformation. Vimentin IFs are responsible for the structural properties of cells as well as functional processes. For example, these proteins control signaling pathways, inflammatory responses and even metabolism (Herrmann and Aebi, 2016; Ridge et al., 2022).

*Solanum aculeastrum* Dunal fruit extract, containing SM and SN, had a cytotoxic effect and inhibited the activity of P-glycoprotein in both neoplastic and healthy cells. SH-SY5Y NM cells (NM) were more susceptible to the effects of both the crude extract and its water fraction; cytotoxic effects were observed, and inhibition of P-glycoprotein activity was caused by the action of SM (Burger et al., 2018).

Wild potato leaf extract *Solanum chacoense* Bitt., containing solasodine, SM, SN and CH, exhibited selective cytotoxic properties for MCF7 breast cancer BC cells compared to healthy HUVECs (human umbilical vein endothelial cells). Both the leaf extract and the tuber extract of this plant increased the expression of the pro-apoptotic Bax gene and decreased the expression of the anti-apoptotic Bcl-2 gene and genes promoting the proliferation of NFκB, CCND1, and STAT3 cells. For this reason, GAs or other substances such as phenolic acids may be responsible for the properties of these extracts (Cruceriu et al., 2021).

Actinic keratosis is a pathological change that predisposes patients to squamous cell carcinoma of the skin. SR-T100 is a newly patented drug extracted from *Solanum incanum* L. It contains SM as an active ingredient and is used in the form of a gel. In the third phase of clinical trials, the effectiveness and safety of its use in the treatment of actinic keratosis was demonstrated (Yang et al., 2018). This drug increases the expression of tumor necrosis factor and activates the mitochondrial apoptosis pathway. The IC_50_ for SR-T100 is higher in OC cells than in healthy cells. The drug decreased the expression of aldehyde dehydrogenase 1 (ALDH1), Notch1, FoxM1, c/EBPPβ and *COL11A1* genes. Furthermore, SR-T100 suppressed the growth of A2780CP70 cells in mouse xenografts and, in combination with cisplatin, increased the effectiveness of ovarian cancer treatment (Wu et al., 2015). SR-T100 has been shown to induce apoptosis, DNA degradation, and cell cycle arrest in the G0/G1 phase in murine B16 MN cells. Additionally, in *in vivo* studies in mice, intravascular injection of the drug reduced tumor size, and intraperitoneal injection inhibited LC growth and the number of metastases (Yu et al., 2017).

The extract of *S. melongena* L. peel applied twice a day for 12 weeks reduced the diameter of skin lesions in arsenic-induced Bowen disease (Sarah and Misbahuddin, 2018).

SN is one of the four components of the SANT mixture, which contains active ingredients of traditional Chinese botanical drugs that exhibit anticancer effects. The potential of SANT in treating heparanase (HPSE)-related triple-negative breast cancer (TNBC) was identified *in vitro* and *in vivo*. HPSE is a glucuronidase responsible for the resistance of tumor cells to chemotherapy. It promotes proliferation, invasion, metastasis, and angiogenesis of the tumor. SANT upregulates the expression of genes encoding ATG16L1, ATG9B, and ATG4D while downregulating TMEM74 and TNF gene expression. Moreover, it decreases the levels of HB-EGF, thrombospondin-2, amphiregulin, leptin, IGFBP-9, EGF, coagulation factor III and MMP-9 proteins but increases serpin E1 and platelet Factor 4 levels. The mixture of botanical drugs also reduces tumor growth and angiogenesis *in vivo* (Li et al., 2021b).

To enhance the anticancer effect of GAs, high temperature may be used. Research shows that the effect of high temperature on *S. tuberosum* L. juice increases the antioxidant potential and cytotoxic properties toward Caco-2 and HT-29 intestinal cancer (IT) cells. At the same time, the cytotoxicity of this extract for nonmutated cells is the same or lower than the cytotoxicity of fresh juice. Thermal deproteinization does not affect the content of GAs in the juice, while spray-drying decreases in its content. Low temperatures do not affect the GA content and do not increase the cytotoxic properties against neoplastic cells (Kowalczewski et al., 2019).

Furthermore, the extract of SM and SS from *S. lycocarpum* A.St.-Hil. has been shown to cause chemosensitization of RT-4 BC cells to cisplatin chemotherapy, indicating a synergistic effect of both substances. PDX cells (patient-derived xenografts) were characterized by much higher resistance to cisplatin than RT-4 cells. PDX xenografts are human models composed of cancer cells introduced into the body of immunodeficient mice and are used to test anticancer drugs. The extract in combination with cisplatin induced apoptosis and inhibited the growth and migration of RT-4 cells. The mechanism of apoptosis induced by cisplatin and GA extract is a decrease in the expression of genes encoding anti-apoptotic proteins Bcl-2, Bcl-xL, survivin, and PARP and increase in the expression of genes encoding the pro-apoptotic protein Bax and activate caspases. Proteins of the PARP family (poly (ADP-ribose) polymerase) are responsible for the repair of damaged DNA; therefore, excessive expression of the genes encoding them may make cancer cells resistant to treatment. Furthermore, a decrease in the expression of MMP-2 and MMP-9 metalloproteinases was observed, indicating the effect of this combination therapy on cell invasion and migration (Miranda et al., 2021).

Because of the poor solubility of GAs in water and low bioavailability, delivery of GA to cancer cells is an issue. An-te-xiao capsules containing alkaloids obtained from whole dried *S. lyratum* Thunb. plants had an inhibitory effect on NSCLC cells from the A549, NCI-H460 and NCI-H520 lines *in vitro* and *in vivo*. These capsules significantly extended the life of mice with Lewis lung cancer cells (xenografts). An-te-xiao influences tumor growth, microvessel density, cell cycle regulatory proteins, and apoptosis and causes histopathological changes in neoplastic cells. *In vitro* studies showed that the capsules inhibited migration, invasion, and vessel formation in Td-ECs (tumor-derived endothelial cells) both in the presence and absence of VEGF. Additionally, they decrease VEGF secretion by A549 lung adenocarcinoma cells and phosphorylation of this growth factor receptor (Han et al., 2018). GA nanocapsules with a diameter of 125 ± 6 nm were also created using polylactic acid, and the cytotoxic effect of the *S. lycocarpum* extract was observed in RT-4 BD cells. Apoptosis and blocking of the cell cycle in the S phase were demonstrated; therefore, the use of nanocapsules may overcome the poor solubility of GAs in water (Miranda et al., 2019). The use of natural lipid-based nanoparticles (myristyl myristate, illipe butter) also decreased the viability of bladder cancer cells by 5.4 times (Carvalho et al., 2019). Additionally, nanoparticles with a particle size of 177 nm functionalized with folate were developed and used against BC MDA-MB-231 and BD RT4 cancer cells. The IC_50_ for these nanoparticles was lower than that of the free extract, indicating that they can penetrate cancer cells. Furthermore, nanoparticles have greater cellular uptake in cancer cells than healthy cells (Miranda et al., 2020). These results suggest that the use of GA-loaded nanoparticles is promising in cancer treatment and could increase the therapeutic efficacy of applied glycoalkaloids.

## Conclusion and future perspectives

*Solanaceae* GAs seem to be a promising agent for the development of alternative methods of cancer treatment. This study summarizes the latest *in silico*, *in vitro,* and *in vivo* studies on the anticancer properties of these compounds, with a focus on their mechanisms of action. These plant secondary metabolites have the potential to become new pharmaceuticals for the treatment of tumors because they act on different cancer cell lines, even at low concentrations. GAs are involved in many cell signaling pathways; therefore, they can influence cancer cells through diverse mechanisms, such as interactions with microtubules or changes in Bax and Bcl-2 expression. These compounds may be applied separately or in combination with other drugs or therapies, improving their effectiveness. GA extracts from plants also have promising cytotoxic effects on tumor cells due to the synergistic effects of their components. Key factors that influence the anticancer activity of GAs include the location, type, and number of carbohydrate molecules. There is a strong relationship between structure and function; therefore, modification of GA structures may also allow for the discovery of new potential anticancer drugs (Akter et al., 2015; Ahmad, 2019). However, some studies indicate that cytotoxic effects due to these compounds also occur in healthy cells. Therefore, in the search for new directions for the use of GAs, it is important to consider the safety of GA drugs. Taken together, although the effects of GAs on cells have been intensively studied, the precise mechanisms of their action in organisms remain to be elucidated. More research needs to be conducted to explore new, more selective, and less toxic cancer therapies and improve the effectiveness of applied treatment methods because cancer remains one of the leading causes of death worldwide.
